# Promoting independence, health and well-being for older people: a feasibility study of computer-aided health and social risk appraisal system in primary care

**DOI:** 10.1186/s12875-017-0620-6

**Published:** 2017-03-24

**Authors:** Kate Walters, Kalpa Kharicha, Claire Goodman, Melanie Handley, Jill Manthorpe, Mima Cattan, Steve Morris, Caroline S. Clarke, Jeff Round, Steve Iliffe

**Affiliations:** 1 Department Primary Care & Population Health, University College London (UCL), Royal Free Campus, Rowland Hill St, London, NW3 2PF UK; 2 Centre for Research in Primary and Community Care, University of Hertfordshire, College Lane, Hatfield, AL10 9AB Hertfordshire UK; 3 Social Care Workforce Research Unit, King’s College London, Strand, London, WC2B 4LL UK; 4 University of Northumbria, Sutherland Building Newcastle-upon-Tyne, Newcastle, NE1 8ST UK; 5 Department of Applied Health Research, UCL, 1-19 Torrington Place, London, WC1E 7HB UK

**Keywords:** Ageing, Elderly, Primary Care, Health Promotion

## Abstract

**Background:**

With population ageing, research is needed into new low-cost, scalable methods of effective promotion of health and wellbeing for older people. We aimed to assess feasibility, reach and costs of implementing a new tailored computer-aided health and social risk appraisal system in primary care.

**Methods:**

Design: Feasibility study.

Setting: Five General Practices in London (Ealing) and Hertfordshire, United Kingdom (UK)

Participants: Random sample of patients aged 65 + years.

Intervention: The Multi-dimensional Risk Appraisal for Older people (MRA-O) system includes: 1) Postal questionnaire including health, lifestyle, social and environmental domains; 2) Software system generating a personalised feedback report with advice on health and wellbeing; 3) Follow-up of people with new concerning or complex needs by GPs or practice nurses.

Evaluation: Feasibility of implementation; participant wellbeing, functional ability and quality of life; social needs, health risks, potential lifestyle changes; and costs of implementation.

**Results:**

Response rates to initial postal invitations were low (526/1550, 34%). Of these, 454/526 (86%) completed MRA-O assessments. Compared to local UK Census data on older people, participants were younger, more were owner-occupiers and fewer were from ethnic minority groups than expected. A range of problems was identified by participants, including pain in last week (269/438, 61.4%), low physical activity (173/453, 38.2%), sedentary lifestyle (174/447, 38.3%), falls (117/439, 26.7%), incontinence (111/441 25.2%), impaired vision 116/451 (25.7%), impaired hearing (145/431, 33.6%), depressed mood (71/451, 15.7%), impaired memory (44/444 9.9%), social isolation (46/449, 10.2%) and loneliness (31/442, 7.0%). Self-rated health was good/excellent in 312/437 (71.4%), and quality of life and well-being were slightly above age-specific population norms. Implementation costs were low. Practices reviewed medical records of 143/454 (31.5%) of participants as a consequence of their responses, and actively followed up 110/454 (24.2%) of their patients.

**Conclusions:**

A computer-aided risk appraisal system was feasible for General Practices to implement, yields useful information about health and social problems, and identifies individual needs. Participation rates were however low, particularly for the oldest old, the poorest, and ethnic minority groups, and this type of intervention may increase inequalities in access. Widespread implementation of this approach would require work to address potential inequalities.

## Background

With increasing life expectancy major challenges face governments internationally [1], and the care and support of older people are widely recognised as a public health imperative. Current policy emphasises the importance of personalised preventative approaches in community settings [2–4]. There is, however, a limited evidence base about the most appropriate approaches to meet these broad aims. Complex interventions designed to improve physical function and maintain independent living show potential to alter health behaviours, improve general health perception and self-efficacy, and promote independence in older people [5].

An ideal multi-faceted approach to promote healthy ageing in the general population of older people should be low cost, easy to implement using existing structures, and demonstrate effectiveness in improving wellbeing in later life. In the UK the majority of older people are registered with a General Practitioner (GP), and primary care is one of the few services that has the ability to reach the general population of older people. There is a lack of evidence for effective primary care led population approaches to improving health and wellbeing in later life, and initiatives such as an annual over 75 years health check, generally an invitation to a face to face assessment with a nurse or physician for all people aged 75 and over, have had limited effectiveness [6], with no evidence on whether they might be cost-effective. This approach is potentially burdensome for primary care, particularly in the context of a rising older-old population. It may also miss opportunities for health promotion in younger age groups, such as those aged 65 – 74 years around the retirement window.

The Health Risk Appraisal for Older people (HRA-O) system is a health risk assessment tool focussed on risk factors for functional decline [7–9]. In this system older people are invited to complete a comprehensive survey on their health and wellbeing across different domains, and then receive an individualised report generated from their answers by a dedicated computer programme. If this report is also shared with primary care practitioners, those with more complex or concerning needs can be actively followed up for further face to face assessments. This process can be automated, using editable software, and the report can include healthy ageing advice, signposting to local and national resources, and be tailored to individuals based on their responses. It can create opportunities for promoting self-care and wellbeing, and has the capacity to integrate social and health care information for older people. Aggregated data have the potential to contribute to the development of profiles of local populations and their needs, and so refine and inform commissioning of services [10].

The pilot and feasibility studies of the HRA-O system in primary care showed that the technology can be culturally adapted to the UK, is acceptable to older people and GPs, and can identify under-utilisation of preventive services [7, 8]. A Randomised Controlled Trial (RCT) of HRA-O in the UK confirmed its acceptablity and had high response rates, but, without any local embedding of its use or reinforcement of its messages by active follow-up through primary care, its impact on changing health behaviour was limited [9]. However in Switzerland an RCT of the HRA-O, linked to active telephone follow-up by nurses and primary care physicians, led to signficant improvements in healthy behaviours after 2 years, and reduced mortality over 8 years [11]. Further development work to expand the HRA-O system to include social, economic and environmental factors determining well-being was undertaken in the UK in the Smarter Working in Social & Health Care project (SWISH) [10, 12]. In this study older people prioritised and developed a series of supplementary questions and feedback on housing, transport, income maximisation, safety, social isolation, care responsibilities, recent life events, access to services and environmental concerns, to add to standard Heath Risk Appraisal (HRA). We have combined the standard HRA-O with the new questions from the SWISH study to create a new broader assessment tool, the Multi-dimensional Risk Appraisal for Older people (MRA-O) system.

This experience raised two questions: 1) Can a multi-faceted intervention with local embedding and re-enforcement of tailored recommendations be integrated into routine primary care? 2) Are there added benefits to broadening primary care based health risk appraisal to include social, economic and environmental domains? The aims of this study were to test the feasibility and costs of using HRA-O and SWISH tools combined into a Multi-dimensional Risk Appraisal system for Older people (MRA-O), including local embedding and re-enforcement of use in routine primary care.

## Methods

### The intervention

The complex intervention had 2 stages:

#### Stage 1) Locally adapting and embedding the MRA-O technology

Research staff facilitated a working group at each of two sites (see below) including 10–14 local representatives of older people, Public Health, GPs, Community Nursing, Adult Social Care, Local Council, Private Sector Providers, Voluntary and Community Organisations, to tailor the MRA-O system to local needs, systems and services. The locality working groups identified local agencies and individuals who could support older people in meeting individual needs identified using the MRA-O system. Supplementary web-searches were conducted, and resources/services identified and contacted by telephone, to collect up to date information on local services for older people in each area. This information was incorporated into the MRA-O software.

#### Stage 2) Feasibility of implementing the MRA-O in primary care

The locally adapted MRA-O system was tested by conducting assessments using postal questionnaires with a random sample of older people registered with participating General Practices. Data were entered and automated personalised feedback was provided to individuals including details of local and national resources to support self-care and promote wellbeing. A copy of the feedback report was scanned into participating patients’ GP health records and reviewed by a lead clinician in each practice (practice nurse, nurse practitioner, or GP). Those with complex or concerning needs had, with consent, additional follow-up from clinicians in primary care (practice nurse, nurse practitioner or GP) to encourage them to engage with actions to promote wellbeing. Practices were advised that the lead clinician should review the MRA-O report on arrival as with any clinical report they receive about patients, and decide on action needed with reference to the medical records, taking into account prior knowledge of the patient. They were given a flow-chart with brief instructions on the process, advised to follow-up those with new, complex and/or concerning needs, and given examples of these (e.g. recent unintentional weight loss, depression, severe pain, loneliness or multiple problems across several domains).

### Study setting

We conducted the study in two localities (London Borough of Ealing, and the County of Hertfordshire, UK) representing urban and semi-rural locations, with different proportions of black and minority ethnic (BME) populations and different socio-economic characteristics. Two General Practices participated in Hertfordshire, and three in Ealing.

### Study sample

A random sample of up to 1 in 3 community dwelling older people (aged 65 years and over) registered with participating General Practices were approached to complete assessments as described in Stage 2 above. The sampling ratio was determined according to the numbers of older people on the practice list, in order to achieve a target sample of 150–200 older people per practice, with an estimated response rate of 40%. We excluded those: living in nursing or residential care settings; with severe incapacitating/life-threatening/terminal illness; unable to provide informed consent (e.g. with moderate to severe dementia/learning disability); where an assessment would be considered burdensome at the time of recruitment (e.g. very recent bereavement or life-threatening illness).

### Data collection

GPs wrote to all randomly selected eligible older people, inviting them to participate in the study. Those consenting to participate were sent a comprehensive (28-page) postal questionnaire survey. One postal reminder was sent to non-responders. All materials were produced in large print versions. Translated versions of the study information sheet and consent forms were available for the main other languages at study sites. The main questionnaire was in English only, and participants were encouraged to seek support from friends or family in completion if needed. A researcher assisted (by telephone) anyone who was unable to complete the written questionnaire due to, for example, sight, language or literacy problems. Data from the questionnaires were entered into the dedicated software programme by the research team.

### Measurements

#### Socio-demographic characteristics

We collected data on age, gender, housing tenure, housing type, age of completion of full-time education, country of birth, English as first language, self-reported ethnic group, receipt of pension, receipt of financial benefits, household composition, marital/civil partnership status, previous main employment and current employment/volunteering. We also asked about mobile telephone use (regular/occasional/none) and internet use (most days/1-3 days per week/< once weekly/never).

#### Feasibility

We collected data on uptake and compared the socio-demographic characteristics of those participating with UK 2011 Census data for that local area [13], where available. We recorded data on actions from practices following MRA-O assessments, including review of medical records, and consequent follow-up and referrals under-taken.

#### Quality of life, health behaviours and well-being

The MRA-O includes: a combination of the HRA-O [14] and additional questions generated from the SWISH study [12]. The HRA-O covers domains of physical health, functioning/activities of daily living, disability, psychological distress, memory and lifestyle behaviours, eg physical activity, nutrition, smoking, use of alcohol and problem alcohol drinking (the CAGE questionnaire [15]) and the four Fried questions (Changed walking half a mile/Walk half a mile less often/Changed climbing 10 steps/Climb 10 steps less often), which predict disablement [16]. Questions from the SWISH study [12] included were on housing, transport, income maximisation, safety, social isolation, care responsibilities, recent life events, access to services, and environmental issues. A supplementary questionnaire included measures of quality of life (SF-12) [17], well-being (Warwick-Edinburgh Mental Well-being Scale 14-item) [18] and health, social care and voluntary sector service use.

#### Cost of the intervention

Data were collected on work required from practices to complete the screening of practice lists, invite patients to participate and to perform patient assessments. Unit costs were estimated for all activities using published unit costs of health and social care activity [19]. From this data we estimated the direct costs of the intervention.

### Analysis

Data from the MRA-O software were generated as Excel files and transferred to SPSS. Other data were directly entered in SPSS. Data were double entered and discrepancies resolved. Data were cross-checked by tabulations and histograms identifying missing data and anomalies and cleaned with reference to the original questionnaires. Descriptive data analysis was undertaken only, as the primary purpose of this study was to determine the feasibility of implementing this process.

## Results

### Response rates

We approached 6 practices to participate and 5 agreed, 2 in Hertfordshire and 3 in Ealing, with an average list size of 8,142 (range 4,264 – 10,231) registered patients. Of the 5 practices, 3 had not previously participated in any research. Response rates to initial postal invitations to older people to participate were low (526/1550, 34%). Of those initially expressing an interest, 454/526 (86.3%) returned full completed questionnaires. There were differences across sites, with 283/770 (36.8%) returned in Hertfordshire and 171/780 (21.9%) in London. Two participants contacted us for support in completing the questionnaire, and for one person to our knowledge the questionnaire was translated by and completed with her son as the participant could not read English.

### Socio-demographic characteristics of participants

Characteristics of participants by study site are shown in Table 1. Overall the median age of participants was 73.2 years (IQR 68.9-78.9 years), 53% were female, 385/447 (86.1%) were White British ethnicity, 403/447 (90.2%) lived in owner-occupied accommodation, 274/451 (60.8%) had no post-16 years education, 118/454 (26.0%) only received a state pension. Compared to local UK 2011 Census data, participants were younger, more were owner occupiers and fewer were from ethnic minority groups than expected. UK 2011 Census data [13] showed that in Ealing’s older population (65 and over) 13% were aged 85 or more, 55% were female, 69% were home owners, 33% lived alone and 37% were from Black, Minority Ethnic (BME) groups. In Hertfordshire, census data showed that 14.7% were over 84, 51% were female, 71% home owners, 28% lived alone and 9% were from BME groups.

**Table 1:** Characteristics of participants, by setting

Characteristic^a^	Urban (Ealing) n (%) N = 171	Semi-rural (Hertfordshire) n (%) N = 283	Overall n (%) N = 454
Age group			
65-74 years	94 (55.0%)	178 (62.9%)	272 (59.9%)
75-84 years	63 (36.8%)	88 (31.1%)	151 (33.3%)
85+ yrs	14 (8.2%)	17 (6.0%)	31 (6.8%)
Female	108 (63.2%)	132 (46.6%)	240 (52.9%)
Born outside UK (missing = 4)	60 (35.7%)	12 (4.3%)	72 (16.0%)
Self-reported Black & Minority Ethnic Group (missing = 7)	53 (31.7%)	9 (3.2%)	62 (13.9%)
Living alone (missing = 5)	53 (31.6%)	60 (21.4%)	113 (25.2%)
Married or equivalent (missing = 8)	93 (56.4%)	209 (74.6%)	302 (67.9%)
Owner occupier (missing = 7)	148 (89.2%)	255 (90.8%)	403 (90.2%)
Age completed education (missing = 3)			
16 years or less	74 (44.1%)	200 (70.7%)	274 (60.8%)
17 years or more	94 (56.0%)	83 (29.3%)	177 (39.3%)
Currently employed (missing = 8)	20 (12.0%)	30 (10.7%)	50 (11.2%)
Volunteering (missing = 7)	54 (32.1%)	57 (20.4%)	111 (24.8%)
Mobile phone use (missing = 10)			
Never	19 (11.7%)	27 (9.6%)	46 (10.4%)
Occasional	79 (48.4%)	147 (52.3%)	226 (50.9%)
Regular	65 (39.9%)	107 (38.1%)	172 (38.7%)
Internet use (missing = 9)			
Never	59 (35.8%)	104 (37.1%)	163 (36.6%)
Occasionally (<once a week)	13 (7.9%)	22 (7.9%)	35 (7.9%)
Sometimes (1-3 days a week)	20 (12.1%)	31 (11.1%)	51 (11.5%)
Often (4 or more days a week)	73 (44.2%)	123 (43.9%)	196 (44.0%)

^a^Data missing for some participants as indicated

## MRA-O findings

### Health problems (physical and psychological), life style and functioning

Table 2 shows health problems identified by participants. Overall health was reported as poor or fair in more than a quarter (125/437, 28.6%). Pain in the last week was very high in this sample (269/438 61.4%), as was pain that limited activity or disturbed sleep (160/429, 37.3%). Sensory deficits (vision and hearing) affected a quarter to a third of the sample. Just over a third of participants reported sedentary behaviour, defined as self-reported sitting for 4 or more hours daily (not including sleep periods). A quarter to a third of participants reported a change in walking half a mile or climbing stairs in the last year. There was a low prevalence of smoking at 5.1%, but the majority (84%) reported a high fat diet. A small proportion (6.8%) screened positive for a possible alcohol problem using the CAGE questions [15].

**Table 2:** Prevalence of health, impairment and lifestyle risks

Item^a^	n (%) N = 454
General health	
Fair or poor self-rated health (missing = 17)	125 (28.6%)
Experienced pain in last 7 days (missing = 16)	269 (61.4%)
Pain causing impairment to activities or sleep (missing = 25)	160 (37.3%)
Physical activity	
Low level of physical activity (missing = 1)	173 (38.2%)
Not able to walk 1 km/½ mile (missing = 10)	70 (15.8%)
*In those able to walk 1 km/½ mile:*	
Difficulty walking 1 km/½ mile due to health (missing = 46)	63 (16.8%)
Changed walking 1 km/½ mile in last year (missing = 45)	101 (27.0%)
Walk 1 km/½ mile less often in last year (missing = 43)	95 (25.4%)
Cannot climb up 10 steps (missing = 6)	18 (4.0%)
*In those able to climb up 10 steps:*	
Difficulty climbing up 10 steps due to health (missing = 45)	95 (22.1%)
Changed climbing 10 steps in last year (missing = 42)	165 (38.4%)
Climb 10 steps less often in last year (missing = 42)	104 (24.2%)
Sitting 4+ hours/day (missing = 7)	174 (38.9%)
Functional ability	
Poor self-rated eyesight (rated fair, poor, very poor or blind) (missing = 3)	116 (25.7%)
Poor self-rated hearing (rated fair, poor, very poor or deaf) (missing = 23)	145 (33.6%)
Need help with IADLs (missing = 12)	105 (23.8%)
Need help with BADLs (missing = 5)	31 (6.9%)
Fallen in last 12 months (missing = 15)	117 (26.7%)
Urinary incontinence in last year (missing = 13)	111 (25.2%)
Of these 111, those incontinent at least 6 days in last year	81 (73.0%)
Psychosocial impairment	
Depressed mood (missing = 3)	71 (15.7%)
Subjective memory problems (missing = 10)	44 (9.9%)
At risk of social isolation (missing = 5)	46 (10.2%)
Fear of falling (missing = 10)	105 (23.6%)
Lifestyle risks	
Current smoker (missing = 7)	23 (5.1%)
Alcohol problem screening (CAGE) score 2+ (missing = 13)	30 (6.8%)
High fat diet (missing = 9)	375 (84.3%)

^a^Data missing for some participants as indicated

### Social problems

A range of social problems was identified by participants (see Table 3). One in five reported difficulties in maintaining the upkeep of their home, and many (179/422, 42.4%) stated they were unsure if they were claiming all the benefits to which they were entitled. A substantial minority (188/453, 41.5%) reported being badly affected by a recent life event. Around 16% (73/443) had caring responsibilities for others. A relatively low number (31/442, 7.0%) reported that they were lonely much of the time. A few (39/446, 8.7%) were concerned that others might take advantage of them financially; this broad category included nuisance (scam) telephone calls.

**Table 3:** Prevalence of reported social problems

Problem^a^	n, % sample size = 454
Badly affected by recent life event (missing = 1)	188 (41.5%)
Upkeep of home difficult (missing = 10)	99 (22.3%)
Transport difficulties limit activity (missing = 8)	58 (13.0%)
Environment reduces quality of life (missing = 13)	32 (7.3%)
Environment limits activity (missing = 10)	24 (5.4%)
Unsafe in home (missing = 17)	20 (4.6%)
Difficult to keep home warm (missing = 7)	20 (4.5%)
Difficulty paying bills (missing = 7)	29 (6.5%)
Difficulty managing money (missing = 8)	18 (4.0%)
Concerned others might take/have taken advantage financially (missing = 8)	39 (8.7%)
Unsure if getting all benefits (missing = 32)	179 (42.4%)
Caring for another (missing = 11)	73 (16.5%)
Lonely much of the time (missing = 12)	31 (7.0%)

^a^Data missing for some participants as indicated

### Quality of life and wellbeing

The mean quality of life scores were 53.2 (n = 412, SD8.6) on the mental component of the SF-12 and 43.9 (n = 412, SD 12.4) on the physical component. This is slightly higher (better health) than normative population values reported for this age group in the UK [20]. The mean wellbeing score on the Warwick-Edinburgh Mental Well-being scale was 54.3 (n = 407, SD 9.2), slightly above age-specific population norms (mean 51.6, SD 8.71) [21].

### Feasibility: Practice follow-up and workload

**Fig. 1:**
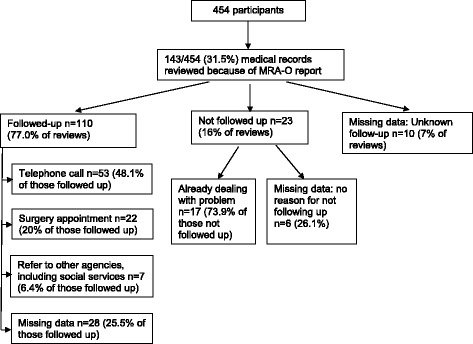
Flow chart of practice follow-up of MRA-O reports

Practices developed systems for dealing with new needs identified by the MRA-O questionnaire using the guidance described in Stage 2 of the intervention above. Overall 110/454 (24%) of participants were followed up by their practice nurse or nurse practitioner (in four practices) or GP (in one practice) by telephone and/or face to face consultation. The numbers followed up varied considerably between practices (range 10%–83%). Figure 1 shows the practice follow-up process and its outcomes.

### Costs of MRA-O use

The costs relate to central administrative tasks and costs for individual GP practices to deliver the intervention. The central administrative task was to locally adapt the MRA-O software to integrate information on local services for needs identified by the MRA-O. We estimate this took up to two days of a junior administrator’s time to verify and update the local contact information for a local directory of services, then enter this in the software. This would need to be updated annually, however is likely to take less time in future years.

The intervention costs of the study for individual GP practices comprised fixed and variable components. The fixed costs relate to the set-up of the intervention. This was £125 (196 USD) per practice in the first year, and then £70 (110 USD) per practice in subsequent years. This covered the cost of identifying patients, including manually reviewing lists generated from electronic searches of GP registers to remove people who were ineligible. The variable costs related to the number of questionnaires sent, returned and requiring following up. These estimates include the costs of the postal questionnaire, data entry, report generation and checking, the time needed to review reports and carry out follow-up by nurse or GP. Data entry was estimated as 11-12 min per questionnaire, which could be performed by a junior administrator, and could be undertaken centrally or within practices.

This cost was £6.71 (10.50 USD) per patient for every 1,000 patients receiving the questionnaire, based on 300 returning the questionnaire (30% response rate) and 24% of those responding requiring follow-up by the GP practice. This cost estimate excludes local adaption of the MRA-O software (stage 1), and refers to costs for using the MRA-O in General Practices. Costs are estimated in 2013 values.

## Discussion

### What this study shows

The MRA-O system functions as a ‘virtual health check’ with the added advantage of measures of social well-being. Unlike other approaches to risk assessment, the provision of computer generated personalised feedback and identification of solutions and services to address identified problems can facilitate self-management of the changes that occur with ageing. Its use is greater amongst those who are owner-occupiers, from white ethnic groups, and the ‘younger old’ age groups (65–84 years). This is similar to previous research [22]. This self-selection bias is likely to underestimate the prevalence of health and social problems in the older population.

Participants nonetheless reported a wide range of health risks and social problems, suggesting that it was not just reaching the ‘worried well’. On reviewing the reports generated, the practice clinician (nurse or GP) needed to review the notes for just under a third of participants, and initiated further follow-up (generally a telephone call or appointment with the nurse or GP) in around a quarter of participants. This additional workload was feasible for practices to undertake as part of their usual care, without needing additional resources or support.

### Comparison with other literature

A wide range of health and social problems was identified, a number of which were at similar levels to those expected, while others were more or less prevalent than in previous research. The proportion of participants who were sedentary is similar to the 40% in the ProAct65+ trial of a health promotion intervention (exercise) for older people in primary care [23]. Sedentary behaviour is associated with adverse physical and mental health outcomes [24], has deleterious health effects, even when physical activity recommendations are met [25], and sitting time is now recognised as a health risk factor independent of physical activity [26]. Older adults are most likely to be sedentary [27, 28]. Almost one in four participants aged 65 and over expressed fear of falling, again similar to other studies in similar populations [29].

In some domains very high prevalence rates were reported. The most striking of these was pain, where more than a third of the sample reported pain limiting their activity or sleep, and more than half had experienced pain during the last week. This is similar to the prevalence of pain identified using the same instrument in an earlier study [30]. Also notable was the salience of a high fat diet in more than 80% of those participating. This is significant given that the ageing process can be viewed as the cumulative effect of chronic health risks including hyperlipidemia, whilst among independently living older people low nutrient density of the diet and inadequate intakes of protein, vitamins, and minerals are the chief areas of nutritional concern [31]. A quarter to a third of respondents noted that their mobility had worsened in the last year. Self-reported preclinical disability (as captured by the Fried questions) predicts incident falls at 1-year follow-up, independent of other self-reported fall risk factors [32]. This is a group whose falls might be prevented, and whose disablement might be interrupted or delayed by intervention. Self-rated impairments in vision and hearing were higher than prevalence rates reported in other studies [33, 34], with nearly a third reporting hearing impairments and a quarter vision impairments.

Some domains were reported less frequently than expected, for example urinary incontinence was lower than shown in other community studies [35, 36] and subjective memory complaints which are reported to occur in 22 - 50% of those aged 65 and over, and increase with advancing age [37, 38]. Just under 7% of participants declared that they were lonely much of the time, compared with a population prevalence of around 16%; severe loneliness occurs in 7-10% of community dwelling people aged over 65 years [39, 40]. Rates of smoking were low, compared to published national statistics of 13% in those aged 60 and over [41], and risk of harmful drinking as measured by the CAGE questions [19] was lower than the published proportions drinking above the recommended limits (19% of men, and 9% of women, aged 65 and over) [42].

### Strengths & limitations

This study reports the feasibility of the implementation of computer-aided health and social risk appraisal for the general population of older people (the MRA-O). This is a novel system that produces a personalised report on both health and social concerns, integrated into routine primary care. The MRA-O itself was developed using co-design methods with significant input from older people, and has undergone extensive and iterative field testing. The implementation study was undertaken in five UK General Practices across two contrasting neighbourhoods in urban London and semi-rural Hertfordshire, chosen to maximise diversity in setting, and in type of practice including those that do not typically take part in research. The findings may not, however, be generalizable to other settings or practices. There was good interest and engagement in participating in the study from General Practices, despite high workloads and the study being undertaken during a period of several changes to the organisation of healthcare in England.

There was a low response rate to the initial invitations to older people, but a high completion rate for those initially expressing an interest in participating, despite the need to complete a lengthy (28-page) questionnaire. We do not know if our findings on prevalence of health and social risks identified would apply to those not taking part. Comparison to local Census data suggests that the prevalence of health and social risks in participants in our study may be underestimates of true prevalence rates, and some groups are likely to be under-represented in those who took part (those over 85 years, those not owning their own home, and those from BME groups). Potential participants were aware that this was being conducted as part of a research study, and there were lengthy information sheets and consent forms regarding this; it is possible that response rates might be higher if this were part of routine clinical care.

### Implications for research, policy and practice

The MRA-O system is feasible to implement in routine practice in primary care, as a case-finding tool that can identify individual health and social needs and risks in the general population of older people. However participation rates were low, particularly for older age people (85 years+), those of lower socio-economic status and older people from BME groups. Further work is needed to increase participation, addressing these inequalities [22, 43, 44]. Some approaches to increasing general participation, such as offering wider access through using web-based questionnaires, email and mobile applications, while attractive for this type of system and would increase operational efficiencies, may widen these inequalities still further. Our own findings confirm that, even in those participating, their use of the internet and regular use of mobile phone is low, particularly in older age groups, though this may change over time. Other potential options include use of a modified, shorter, more focussed questionnaire and/or distributing the initial questionnaire opportunistically, for example alongside annual influenza vaccination campaigns for older people, in routine consultations, or while waiting for appointments in clinics. It may be necessary to target specific audiences where uptake is lower, such as through social or religious groups for older people from BME groups or community centres and social activities for frailer older people over 85 years, where assistance can be given in person to complete the questionnaires.

We need a greater understanding of the best approaches to reach older people from these groups with lower participation rates to ensure any new general population based service does not introduce further inequalities of access. Our study demonstrates that using the MRA-O system can nonetheless identify a broad range of potential unmet needs and health/social risks in those that take part. Furthermore, busy practices were able to use their existing systems to follow up those that they considered had new, complex or concerning needs. We do not know however whether this leads to improvements in care or wellbeing and functioning for older people over time, or if this approach is cost-effective, and this would need to be tested, ideally in a substantive Randomised Controlled Trial. As needs in older people are dynamic and can accumulate over time, we would anticipate that the assessment process may need to be repeated with intervals. The optimum frequency of assessments still needs to be determined. This would balance potential benefit for patients against additional burden for the practices and what is feasible for them to deliver. In our study we asked practices to invite a random third of their older population, in order to reduce the burden on practices for those with large numbers of older people. On this basis one third could be invited annually to cover the whole population and then the process repeated three yearly.

## Conclusions

A computer-aided health and social risk appraisal system is feasible to implement in General Practice settings and can identify a broad range of health, lifestyle and social concerns, provide tailored advice and sign-post older people participating to local services to meet their needs. However, response rates were not high, and there was some evidence that this type of approach may introduce inequalities in access for the older old (85 and over), those who are poorer and from BME groups. These issues should be addressed before implementation in practice.

## Abbreviations

BME groups: Black and minority ethnic groups
CAGE: The CAGE alcohol screening questionnaire
HRA-O: Health risk appraisal for older people
MRA-O: Multi-dimensional risk appraisal for older people
SF-12: Short-form 12 item version – quality of life measure
UK: United Kingdom
